# Examining the Heterogeneity of Exercise Response Among Sedentary Older Adults: A Descriptive Analysis

**DOI:** 10.1155/jare/6952002

**Published:** 2025-03-18

**Authors:** Grace L. Kulik, Melissa P. Wilson, Catherine M. Jankowski, Lindsay T. Fourman, Kristine M. Erlandson

**Affiliations:** ^1^Department of Medicine, University of Colorado Anschutz Medical Campus, Aurora, Colorado, USA; ^2^College of Nursing, University of Colorado Anschutz Medical Campus, Aurora, Colorado, USA; ^3^Metabolism Unit, Massachusetts General Hospital, Boston, Massachusetts, USA

**Keywords:** exercise, geriatrics, heterogeneity, HIV, inflammation

## Abstract

**Background:** Significant heterogeneity in individual responses to exercise interventions provides an opportunity to identify individuals for whom modifications or adjunct therapies may be necessary. Here, we explore heterogeneity of exercise response among people with HIV (PWH) versus without HIV (control).

**Methods:** The Exercise for Healthy Aging Study enrolled sedentary older PWH and controls (50–75 years old) for a 24-week aerobic and resistance exercise program. Responder groups were categorized based on minimally clinically important differences for cardiovascular (CV), strength, and lean mass (LM) outcomes. Descriptive statistics were used to examine baseline characteristics of the different groups.

**Results:** 32 PWH and 37 controls (age 58 ± 6.5 years) were enrolled. CV nonresponders were more likely to have HIV infection, a greater comorbidity burden, and lower baseline CV fitness. Strength nonresponders had a lower comorbidity burden and greater strength at baseline. Comorbidities were similar across LM responder groups. CV and LM nonresponders had an increase in inflammatory markers from baseline to week 24 compared to decreased inflammatory markers among CV and LM responders.

**Conclusion:** Lower CV fitness and HIV infection was more prevalent among those with poorer exercise response, suggesting that higher intensity or a prolonged duration of aerobic exercise may be required to overcome blunted CV adaptations particularly among PWH. Associations of CV and LM response with inflammatory markers should be further explored to determine if and when blocking inflammation might enhance exercise responses.

**Trial Registration:** ClinicalTrials.gov identifier: NCT02404792

## 1. Introduction

Interventions to preserve and improve physical function with aging is a crucial component of treatment for people with HIV (PWH), particularly among older adults. Exercise safely and effectively improves physical function and quality of life and reduces the risk of cardiovascular (CV) disease, diabetes, and metabolic syndrome, among both the general population and PWH [1–3]. However, there is significant heterogeneity in magnitude and type of responses to exercise training, even within identical exercise programs.

A recent National Institutes of Aging workshop summarized the current state of research on the variation of exercise adaptation among older adults [4]. Several reasons underlying this variation included the use of concurrent pharmacotherapy, chronic inflammation, and history of sedentary behavior, factors which are commonly seen in many older PWH [5, 6]. One key point was that early identification of ‘low responders' to exercise [4], may inform additional therapeutic interventions or alterations to the existing exercise prescription to maximize responses and longer-term adherence. The workshop concluded that further research in this field is urgently needed to better improve outcomes and maximize the health benefits of exercise for all older adults [4].

Individual characteristics underlying the wide variability in response to exercise among PWH are currently unknown. We sought to describe the characteristics among responders and nonresponders to a 24-week supervised exercise program in older adults with and without HIV.

A broader understanding of potential characteristics may aid in earlier identification of those at higher risk of nonresponse, and if HIV status is associated with nonresponse. Ultimately, our goal is to develop personalized exercise programs to maximize functional gains for all older adults.

## 2. Methods

### 2.1. Study Design

This study is a secondary analysis of the Exercise for Healthy Aging Study, a clinical trial that examined the effects of 24 weeks of supervised aerobic and resistance exercise among sedentary older adults with and without HIV.

### 2.2. Participants

The Exercise for Healthy Aging Study enrolled sedentary older adults (50–75 years old) with and without HIV. Participants were defined as sedentary if they reported less than 60 min of physical activity per week, on average, for the prior 6 months. PWH were required to be on stable antiretroviral therapy with viral load < 200 copies/mL and CD4 count greater than 200 cells/μL. Full inclusion and exclusion criteria have been previously published [3].

### 2.3. Exercise Training Intervention

Participants attended in-person, supervised exercise sessions at the University of Colorado-Anschutz Medical Campus three times per week for 24 weeks. Aerobic exercise was performed on a treadmill; resistance exercises (leg press, lateral pulldown, and chest press) were performed with weight machines. A cardiorespiratory exercise test was performed at baseline to measure maximum oxygen consumption (VO_2_ max) and maximum heart rate. The endurance training prescription was based on the maximal heart rate achieved. A 1-repetition maximum (1-RM) test was performed at baseline and every 4 weeks to determine the resistance exercise intensity. Participants were familiarized with the intervention during the first 2 weeks by beginning with low-intensity aerobic (30%–40% of VO_2_ max) for 20–30 min and resistance exercise (40%–50% of the 1-RM). After the first 2 weeks, participants increased aerobic intensity to 40%–50% VO_2_ max and time on the treadmill in 5-min increments until a total time of 50 min on the treadmill was achieved. Resistance exercise was progressed to 60%–70% of 1-RM in the first 3 weeks. At Week 12, VO_2_ max was repeated, and participants were randomized to continue at moderate intensity exercise (40%–50% VO_2_ max) or progress to high intensity for an additional 12 weeks. Further details regarding the intervention have been previously published [3].

### 2.4. Baseline Measures

Demographics were self-reported during the first baseline visit. Medical history and medications were derived from chart review and confirmed with each participant. Adherence was defined as the number of attended exercise sessions, divided by the number of expected sessions. Frailty was defined using Fried's criteria (weakness via handgrip strength, self-reported exhaustion, slow walking speed, unintentional weight loss, sedentary) [7]. Participants were considered nonfrail if they had 0 components, prefrail if they had 1 or 2 components, and frail if 3–5 components were met [7]. The Short Performance Physical Battery (SPPB) was used to define physical function impairment at baseline [8]. The SPPB consists of time to complete 5 chair stands, a 4 m walk at usual speed, and balance testing (semi-tandem and tandem standing) [8]. A score below 12 indicated that there was impaired performance on at least one of the tests, and therefore participants with scores < 12 were categorized as having functional impairment at baseline [3, 8].

Sarcopenia was defined by Baumgartner's criteria (appendicular lean mass [ALM] divided by height in meters squared [ALM/m^2^] < 7.26 for men and < 5.45 for women) [9]. The Veterans Aging Cohort Study (VACS) Index was used as a measure of comorbidity burden [10]. The VACS Index predicts the risk of 5-year all-cause mortality and incorporates age and routine laboratory tests among those with HIV: CD4 count, HIV-1 RNA values, hemoglobin, platelets, liver lab values (AST and ALT), creatinine, and hepatitis C status. Higher scores indicate a higher comorbidity burden [10].

### 2.5. Inflammatory Measures

Serum or plasma inflammatory markers were measured at baseline (interleukin-6 [IL-6], IL-10 [IL-10], high sensitivity C-reactive protein [hs-CRP]), and week 24 (both) as previously described [11]. Area under the curve of acute inflammatory markers (IL-6, soluble tumor necrosis factor receptor 1 [sTNFR1], TNF-α [TNF-a]) were also measured prior to and immediately following exercise (at 0, 60, 90 min) as previously described [11]. Increased levels of IL-6, hs-CRP, sTNFR1, and TNF-a indicated higher levels of systemic inflammation [12]. As an anti-inflammatory cytokine, higher levels of IL-10 were thought to reduce inflammation [13].

### 2.6. Body Composition Measures

Body composition measures (total fat, visceral fat, total lean mass (LM), ALM) were taken from dual-X-ray absorptiometry (DXA) at week 0 and 24 [14]. Body mass index (BMI) was calculated using DXA body mass divided by height squared (kg/m^2^) [14]. DXA is the most commonly used technique for measuring total body composition and is the preferred measurement technique for estimating muscle mass across research and clinical settings [15].

### 2.7. Exercise Outcome Measures

We grouped outcome measures into categories describing different responses to exercise: (1) CV (VO_2_ max, 400m walk time), (2) upper extremity (UE) strength (1-RM bench press, grip strength), (3) lower extremity (LE) strength (1-RM leg press, 10x-chair rise time). The VO_2_ max test was performed on a treadmill, using a protocol where speed was held constant and grade was increased by 2% every 2 min until volitional exhaustion or until the test was stopped. For the 400 m walk test, participants were instructed to walk 8 laps on a 50 m course as quickly but as safely as possible. The 1-RM tests were performed by gradually increasing weight over several sets with decreasing repetitions until the participant could only perform 1 repetition with full range of motion at a given weight. For the 10x chair stand test, participants were timed completing 10 sit-to-stands on a standard height chair without using their arms.

These measures were completed at weeks 0, 12, and 24. For LM response, we examined DXA total appendicular LM normalized to height and total LM, measured at 0 and 24 weeks only. The breakdown of participants that only improved in one outcome per category is reported in the Supporting Information (Supporting Figure S1).

### 2.8. Responder Groups

Two main responder groups were determined using the minimally clinically important difference (MCID) of the outcome measures for older adults found in prior literature. We explored responses across 4 categories: CV [16, 17], UE strength [18, 19], LE strength [19, 20], and LM [21].  Table 1 provides detailed information regarding the MCID threshold for each measure. For each exercise outcome, Responders met or exceeded the MCID of ≥ 1 of the outcome measures during both arms of the study (weeks 0–12 and 13–24). Nonresponders failed to meet (or exceed) the MCID threshold during either half of the intervention. LM was alternatively categorized as: LM responders, LM nonresponders, and LM negative responders. LM responders met or exceeded the MCID, LM nonresponders did not meet or exceed the MCID, and LM negative responders decreased by the MCID, all from baseline to week 24. Groups were further differentiated into Early (improved by ≥ MCID during weeks 0–12 only) and Late Responders (improved by ≥ MCID during weeks 13–24 only; Supporting Tables S1–S3).

### 2.9. Statistical Analysis

Baseline descriptive characteristics are reported as mean (standard deviation [SD]), median (interquartile range [IQR]), or frequency (percentage [%]). Absolute change in the outcome measures were defined as either (week 12–baseline), (week 24–week 12), or for LM outcome measures, (week 24-baseline). Percent changes in those outcomes were calculated as [(absolute change/baseline) × 100]. All analyses were performed in RStudio (version 4.3.2; R Core Team, Vienna, Austria) and graphics were created in GraphPad Prism (version 10.2.0 for Mac OS X; GraphPad Software, Boston, Massachusetts).

## 3. Results

At baseline, 32 PWH and 37 controls were enrolled; 27 PWH and 29 controls completed 24 weeks of training. The mean (SD) age was 58 (6.5) years and participants were predominantly male (93%). Differences in baseline characteristics by HIV status are provided in the Supporting Information (Supporting Table S4). Overall adherence to the exercise program, defined by number of sessions attended, ranged between 82%–88% (Supporting Table S5). Adherence was similar by HIV status (89% of sessions attended by PWH; 86% of sessions attended by controls).

### 3.1. CV Responders

**Figure 1:**
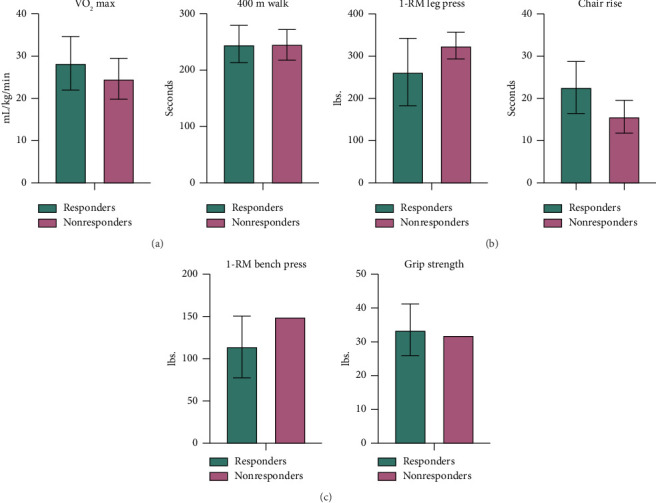
Baseline physiological and physical function measurements for the responder categories (mean (SD)). (a) Cardiovascular outcomes, (b) lower extremity strength outcomes, (c) upper extremity strength outcomes.

**Figure 2:**
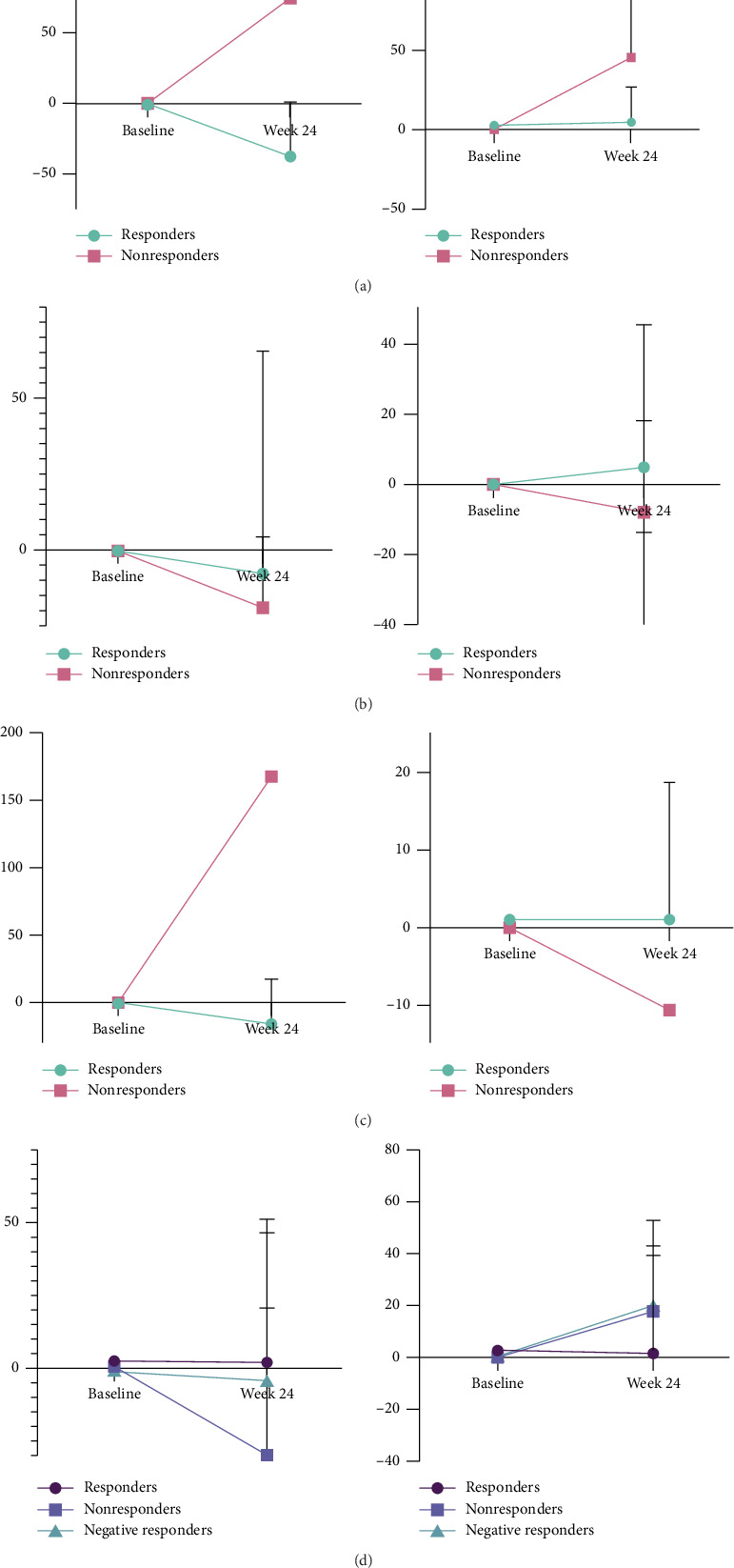
Percent change of inflammatory markers (hs-CRP: left column, IL-6: right column) from baseline to week 24 (median [IQR]). (a) cardiovascular outcomes, (b) lower extremity strength outcomes, (c) upper extremity strength outcomes, (d) lean mass outcomes.

19 participants were classified as CV responders, and 9 were classified as CV nonresponders. As summarized in Table 2, the CV responder group had a younger median age (55 vs. 61 years), and more were randomized to HIT (73% vs. 2%). The CV responder group had a higher baseline VO_2_ max (28.3 vs. 24.6 mL/kg/min) (Figure 1). Nonresponders tended to be PWH (78% vs. 58%), had a higher median baseline VACS score (greater comorbidity burden), and had the lowest exercise adherence. CV responders had a 37% (95% CI: −53.7, 1.6) decrease in hs-CRP and a 1.5% (95% CI: −30.2, 24.3) increase in IL-6 over 24 weeks, while CV nonresponders had a 75% increase (95% CI: 17.9, 110.2) in hs-CRP and 45% increase (95% CI: 23.0, 109.5) in IL-6 (Figure 2).

### 3.2. Strength Response

There were 12 LE strength responders and 7 LE strength nonresponders whose characteristics are summarized in Table 3. The LE strength responder group, although of similar age to LE strength nonresponders, had a higher VACS score, a slower baseline chair rise time, and a lower baseline 1-RM leg press (Figure 1). There was a greater number of PWH categorized as LE strength responders. 4 out of the 5 participants with sarcopenia at baseline also met the criteria for LE strength responder. LE strength nonresponders saw the largest percent decrease in hs-CRP (−18.7%; 95% CI: −32.4, 4.6) and IL-6 (−8.1%; 95% CI: −44.7 18.) from baseline to week 24 (Figure 2). The percentage of participants randomized to HIT was similar between the two groups.

Twenty-three participants met the criteria for UE strength responders and only 1 participant was a nonresponder (Table 4). As a group, the UE responders were slightly older, had a highest VACS Index score, and had a lower grip strength and 1-RM bench press compared to the nonresponder (Figure 1). 4 out of the 5 participants with sarcopenia at baseline met the criteria for UE responder. The one nonresponder had a 168% increase in IL-6 and 200% increase in hs-CRP while the responder group had either decreases or much smaller increases in IL-6 and hs-CRP from baseline to Week 24 (Figure 2).

### 3.3. LM Response

28 participants were LM responders, 16 were nonresponders, and 11 were LM negative responders. The percentage of PWH was similar across the three responder categories (range 46%–50%). LM nonresponders tended to be older, while the LM negative-responders had the lowest comorbidity burden, the greatest mean visceral fat, and largest waist circumference, and were more likely to have been randomized to the HIT arm (Table 5). 10 out of the 11 LM negative responders lost body weight over the course of the intervention. The LM nonresponders also had the largest percent decrease in hs-CRP (−29.9%; 95% CI: −43.3, 20.7) with an increase in IL-6 (+17.7%; 95% CI: −30.4, 39.3) from baseline to week 24 (Figure 2). LM negative responders had the highest percent increase in IL-6 from baseline to week 24 (+21.1%; 95% CI: 15.2, 52.9; Figure 2).

### 3.4. Additional Inflammatory Markers

Baseline inflammatory markers (hs-CRP and IL-6) were similar across all outcomes and all responder groups (Supporting Table S6). There was no observed pattern in IL-10 trends across all outcomes and responder groups (Supporting Table S6). Acute inflammatory responses, measured at baseline up to 90 min following an exercise session, are presented in Supporting Table S7.

## 4. Discussion

The findings from this descriptive analysis provide initial insight into characteristics of exercise responders and nonresponders following 24-weeks of supervised exercise, in a population of people both with and without HIV. Identifying participants that are less likely to respond to exercise and the nature of the responses can allow us to modify or enhance the intervention to maximize responses. We found that CV nonresponders tended to have greater burden from comorbidity including more use of antihypertensive and statin medications, and a lower baseline cardiorespiratory fitness level compared to responders. In contrast, strength nonresponders tended to have less burden from comorbidities, take fewer medications, and have greater levels of baseline strength. Those that lost LM (negative-responders) tended to have lower comorbidity burden but greater visceral fat. Perhaps most interestingly, while groups were quite small, CV and UE nonresponders tended to have the greatest increase in the inflammatory markers hs-CRP and IL-6 during the study, while findings were less consistent in the LE and LM non or negative responders. Lastly, nearly all of the CV nonresponders were PWH, suggesting that this population may need additional interventions to maximize CV response. Several of our findings merit further discussion.

First, the majority CV responder group were randomized to HIT, compared to only 2 of the 9 (22%) in the CV nonresponder group. This is consistent with findings from the HERITAGE Family Study, one of the largest studies to assess CV response to exercise in an adult population [22], where participants who trained at higher intensities relative to their VO_2_ max, had greater improvements in VO_2_ max after training [22] and relative training intensity accounted for most of the variance in VO_2_ max adaptation response. A meta-analysis similarly found that exercise intensity explains the largest variance in VO_2_ max improvement, with the largest improvements seen with training at 66%–73% of maximal heart rate, among previously sedentary older adults [23]. Other studies have suggested that CV response (VO_2_ max specifically) may be blunted among those using statins [24]. The frequency of participants taking statins in the present study were relatively balanced among the CV responder groups (range 42% to 50%); however, we did not distinguish type or dose of statin.

Next, we found that the majority of our participants were classified as strength responders, consistent with previous literature [25]. A retrospective analysis of *n* = 85 older adults found that despite heterogeneity in the magnitude of strength gained following a 24-week supervised exercise program, all participants made positive gains in strength or physical function [25]. Even with our more stringent definition of nonresponder (i.e., improvement by at least the MCID rather than any positive improvement in strength), the present study still only had 1 UE and 7 LE nonresponders. This finding is particularly encouraging among PWH, as older PWH have decreased strength and accelerated decline in physical function compared to uninfected controls. Muscle strength is a key component of facilitating healthy aging and is an independent predictor of all-cause mortality among older adults [26]. Moreover, UE and LE strength nonresponders had the greatest baseline strength, suggesting less room for improvement and likely a need for more advanced strength programming to see further strength improvements. One interesting, albeit surprising, finding was the seemingly opposite trend of inflammation seen among CV and LM responders. LE nonresponders had a decrease in IL-6, compared to responders who increased IL-6. These findings were surprising and inconsistent with our findings in regards to LM (below). However, previous studies have found that the use of anti-inflammatory drugs has no impact on strength improvements or changes; therefore, there may not be a strong association between inflammation and strength changes [27, 28]. Our findings may also be due to the small sample size of strength nonresponders and heterogeneity of inflammatory response.

Maintenance of LM is an essential component of the aging process, particularly among PWH who tend to have faster declines in LM compared to HIV-negative controls [29]. Those who lost LM (negative responders, 20%) had the highest baseline body composition measurements; these participants saw a concomitant loss of LM with loss of weight. We also found that IL-6 increased in the LM nonresponder and negative-responder groups but decreased in the responder group, consistent with literature that shows chronic, low-grade inflammation contributes to attenuated gains in muscle mass [30]. It is unknown whether the exercise program or other lifestyle factors induced these inflammatory changes and subsequently caused a decline in LM, or if the decline in LM is linked to the observed increase in inflammation. In contrast to change in strength cited above, blunting the inflammatory response using nonsteroidal anti-inflammatory drugs with resistance training did result in significantly greater muscle mass in older adults compared to exercise alone [31]. Further studies into mechanisms behind chronic inflammation or strategies (and timing) to blunt persistent inflammation following exercise interventions are warranted.

The present analysis had several limitations. First, the retrospective and descriptive design did not allow for a causal analysis of which factors lead to exercise response or nonresponse. The small sample size distribution between the responder categories, particularly of the nonresponders, was not adequate for statistical comparisons. The MCID values used to stratify the responder groups were derived from other aging populations and MCID values specific to those living with HIV were not available in the literature. However, this preliminary analysis provides insight into patterns of characteristics that may be seen in responders or nonresponders to a 24-week supervised exercise intervention in older adults with and without HIV.

## 5. Conclusion

In summary, this descriptive, exploratory analysis provides information on characteristics of participants with heterogenous responses to exercise, including lower baseline CV fitness among CV nonresponders but greater baseline strength among strength nonresponders. As nearly all of the CV nonresponders were PWH, higher intensity exercise or additional strategies may be required to achieve CV outcomes, particularly among sedentary older adults with lower function and greater comorbid burden. These blunted outcomes could also impact longer term exercise adherence. Lastly, the associations between exercise response and inflammatory changes need further investigation into whether strategies to decrease inflammation or visceral adipose tissue in combination with exercise might enhance exercise responses, particularly among PWH who tend to have chronic, low-grade inflammation. Future research with larger sample sizes should prospectively examine baseline fitness and dosing of exercise programs and explore the association between chronic inflammation and exercise among PWH. A personalized approach to exercise intervention based on initial performance may help to maximize benefits of exercise and promote long-term adherence.

## Figures and Tables

**Table 1:** Description of the minimally clinically important differences.

Measure	MCID threshold
*Cardiovascular responder*	
VO_2_ max (mL/kg/min) [16]	+1.5 mL/kg
400 m walk [17]	−20.0 s

*Upper extremity responder*	
Grip strength [18]	+2.7 kg
1-RM bench press [19]	+20.0%

*Lower extremity responder*	
1-RM leg press [19]	+15.0%
10x chair rise [20]	−3.1 s

*Lean mass responder*	
Appendicular lean mass [21]	+ 2.0%
Whole body lean mass	

*Note:* VO_2_ max: maximal oxygen consumption.

Abbreviation: 1-RM, 1-repetition maximum.

**Table 2:** Characteristics of responders by cardiovascular (CV) outcomes.

	CV responder	CV nonresponder
*n*	19	9
Demographics/social		
Age	55.0 [52.5, 60.5]	61.0 [52.0, 65.0]
Male	17 (89.5)	9 (100.0)
Nonwhite race	2 (10.5)	2 (22.2)
Hispanic ethnicity	5 (26.3)	1 (11.1)
Alcohol use	14 (73.7)	7 (77.8)
Smoking		
Former	9 (47.4)	3 (33.3)
Never or rare	8 (42.1)	4 (44.4)
Current	2 (10.5)	2 (22.2)
Comorbidities/medications		
HIV	11 (57.9)	7 (77.8)
VACS	18.0 [12.0, 24.0]	28.0 [18.0, 33.0]
Pre-frail	12 (63.2)	7 (77.8)
SPPB < 12	5 (26.3)	5 (55.6)
Sarcopenia	2 (10.5)	2 (22.2)
Hypertension	10 (52.6)	6 (66.7)
Hyperlipidemia	11 (57.9)	5 (55.6)
Diabetes	2 (10.5)	0 (0.0)
Antihypertensives	10 (52.6)	6 (66.7)
Statins	8 (42.1)	4 (44.4)
Randomization to HIT	14 (73.7)	2 (22.2)
Overall adherence	88.9 [84.0, 91.7]	81.9 [80.6, 88.9]

*Note:* Data presented in the table is either count (%) or median [IQR].

Abbreviations: HIV, human immunodeficiency virus; SPPB, short performance physical battery; VACS, Veterans Aging Cohort Study Index.

**Table 3:** Characteristics of responders by lower extremity (LE) outcomes.

	LE responder	LE nonresponder
*n*	12	7
Demographics/social		
Age	61.0 [55.5, 65.5]	61.0 [56.5, 63.5]
Male	9 (75.0)	7 (100.0)
Nonwhite race	2 (16.7)	2 (28.6)
Hispanic ethnicity	1 (8.3)	1 (14.3)
Alcohol use	8 (66.7)	6 (85.7)
Smoking		
Former	7 (58.3)	3 (42.9)
Never or rare	5 (41.7)	3 (42.9)
Current	0 (0.0)	1 (14.3)
Comorbidities/medications		
HIV	7 (58.3)	1 (14.3)
VACS	25.0 [12.0, 29.3]	18.0 [12.0, 18.0]
Prefrail	8 (66.7)	3 (42.9)
SPPB < 12	7 (58.3)	1 (14.3)
Sarcopenia	1 (8.3)	0 (0.0)
Hypertension	7 (58.3)	1 (14.3)
Hyperlipidemia	5 (41.7)	1 (14.3)
Diabetes	1 (8.3)	0 (0.0)
Antihypertensives	7 (58.3)	1 (14.3)
Statins	4 (33.3)	1 (14.3)
Randomization to HIT	7 (58.3)	4 (57.1)
Overall adherence	82.6 [77.8, 89.6]	87.5 [86.1, 91.7]

*Note:* Data presented in the table is either count (%) or median [IQR].

Abbreviations: HIV, human immunodeficiency virus; SPPB, short performance physical battery; VACS, Veterans Aging Cohort Study Index.

**Table 4:** Characteristics of responders by upper extremity (UE) outcomes.

	UE responder	UE nonresponder
*n*	23	1
Demographics/social		
Age	59.0 [53.0, 64.0]	57.0 [57.0, 57.0]
Male	21 (91.3)	1 (100.0)
None-white race	1 (4.3)	1 (100.0)
Hispanic ethnicity	2 (8.7)	0 (0.0)
Alcohol use	18 (78.3)	0 (0.0)
Smoking		
Former	10 (43.5)	0 (0.0)
Never or rare	9 (39.1)	1 (100.0)
Current	4 (17.4)	0 (0.0)
Comorbidities/medications		
HIV	12 (52.2)	0 (0.0)
VACS	22.0 [15.0, 28.0]	18.0 [18.0, 18.0]
Pre-frail	15 (65.2)	1 (100.0)
SPPB < 12	8 (34.8)	0 (0.0)
Sarcopenia	4 (17.4)	0 (0.0)
Hypertension	13 (56.5)	1 (100.0)
Hyperlipidemia	14 (60.9)	1 (100.0)
Diabetes	1 (4.3)	0 (0.0)
Antihypertensives	13 (56.5)	1 (100.0)
Statins	12 (52.2)	1 (100.0)
Randomization to HIT	10 (43.5)	0 (0.0)
Overall adherence	87.5 [82.6, 91.7]	87.5 [87.5, 87.5]

*Note:* Data presented in the table is either count (%) or median [IQR].

Abbreviations: HIV, human immunodeficiency virus; SPPB, short performance physical battery; VACS, Veterans Aging Cohort Study Index.

**Table 5:** Characteristics of responders by lean mass (LM) outcomes.

	LM responder	LM nonresponder	LM negative responder
*n*	28	16	11
Demographics/social			
Age	57.0 [53.0, 64.0]	61.0 [55.0, 63.0]	52.0 [51.0, 58.5]
Male	25 (89.3)	15 (93.8)	11 (100.0)
Nonwhite race	3 (10.7)	2 (12.5)	5 (45.5)
Hispanic ethnicity	6 (21.4)	1 (6.2)	1 (9.1)
Alcohol use	22 (78.6)	14 (87.5)	7 (63.6)
Smoking			
Former	11 (39.3)	7 (43.8)	4 (36.4)
Never or rare	16 (57.1)	6 (37.5)	4 (36.4)
Current	1 (3.6)	3 (18.8)	3 (27.3)
Comorbidities/medications			
HIV	14 (50.0)	8 (50.0)	5 (45.5)
VACS	19.0 [12.0, 27.3]	18.0 [12.0, 26.5]	12.0 [12.0, 20.0]
Prefrail	20 (71.4)	9 (56.2)	7 (63.6)
SPPB < 12	9 (32.1)	2 (12.5)	2 (18.2)
Sarcopenia	4 (14.3)	1 (6.2)	0 (0.0)
Hypertension	12 (42.9)	8 (50.0)	5 (45.5)
Hyperlipidemia	14 (50.0)	10 (62.5)	6 (54.5)
Diabetes	3 (10.7)	2 (12.5)	1 (9.1)
Antihypertensives	12 (42.9)	8 (50.0)	5 (45.5)
Statins	12 (42.9)	7 (43.8)	6 (54.5)
Baseline body composition measures			
Total fat (mean (SD))	29.5 (5.9)	25.5 (5.9)	29.6 (6.6)
Visceral fat (mean (SD))	207.0 (76.9)	157.5 (53.6)	232.0 (85.8)
Total lean mass (mean (SD))	58.0 (10.1)	57.3 (4.1)	63.4 (10.3)
ALM (mean (SD))	8.6 (1.2)	8.6 (0.8)	9.3 (1.1)
Waist circumference (mean (SD))	99.1 (13.2)	93.8 (9.2)	106.0 (13.0)
BMI (mean (SD))	28.5 (4.5)	26.6 (3.6)	30.0 (4.5)
Randomization to HIT	13 (46.4)	8 (50.0)	8 (72.7)
Overall adherence	87.5 [81.6, 91.7]	89.6 [86.8, 91.7]	84.7 [75.0, 88.9]

*Note:* Data presented in the table is either count (%) or median [IQR] unless otherwise specified.

Abbreviations: ALM, appendicular lean mass; BMI, body mass index; HIV, human immunodeficiency virus; SPPB, short performance physical battery; VACS, Veterans Aging Cohort Index.

## Data Availability

The data that support the findings of this study are available from the corresponding authors upon reasonable request.

## Supporting Information

The supporting information contains further detail on characteristics of participants by responder group. Supporting tables S1–S3 provide characteristics of participants who responded in only one arm of the study (early or late responders). Table S4 displays baseline demographics by HIV status. Table S5 displays exercise adherence and adverse events. Table S6 and S7 provide further detail on baseline inflammation, change in IL-10, and the acute inflammatory response by responder group. Supporting Figure S1 shows the breakdown of participants who improved in only one outcome.
