# Supplementary material for: Examining the Heterogeneity of Exercise Response Among Sedentary Older Adults: A Descriptive Analysis
Source: J Aging Res. 2025 Mar 18;2025:6952002. doi: 10.1155/jare/6952002 (PMC11936528; doi:10.1155/jare/6952002)
Supplement: Supporting Information — Additional supporting information can be found online in the Supporting Information section. [file 6952002.f1.docx]

**SUPPLEMENTAL MATERIAL**

**Supplemental Table S1.** Characteristics of early and late responders by cardiovascular (CV) outcomes.

|  | **CV Early Responder** | **CV Late Responder** |
| --- | --- | --- |
| n | 20 | 8 |
| **Demographics/Social** |  |  |
| Age | 59.5 [54.8, 62.5] | 59.5 [52.0, 64.8] |
| Male | 18 (90.0) | 8 (100.0) |
| Non-White Race | 5 (25.0) | 1 ( 12.5) |
| Hispanic Ethnicity | 1 ( 5.0) | 1 ( 12.5) |
| Alcohol use | 16 (80.0) | 7 ( 87.5) |
| Smoking |  |  |
| Former | 8 (40.0) | 2 ( 25.0) |
| Never or rare | 11 (55.0) | 4 ( 50.0) |
| Current | 1 ( 5.0) | 2 ( 25.0) |
| **Comorbidities/Medications** |  |  |
| HIV | 8 (40.0) | 1 ( 12.5) |
| VACS | 18.0 [12.0, 25.0] | 15.0 [12.0, 20.3] |
| Pre-frail | 13 (65.0) | 4 ( 50.0) |
| SPPB < 12 | 2 (10.0) | 1 ( 12.5) |
| Sarcopenia | 1 ( 5.0) | 0 ( 0.0) |
| Hypertension | 7 (35.0) | 3 ( 37.5) |
| Hyperlipidemia | 11 (55.0) | 4 ( 50.0) |
| Diabetes | 2 (10.0) | 2 ( 25.0) |
| Antihypertensives | 7 (35.0) | 3 ( 37.5) |
| Statins | 9 (45.0) | 4 ( 50.0) |
| **Randomization to HIT** | 10 (50.0) | 3 ( 37.5) |

**Supplemental Table S2.** Characteristics of early and late responders by lower extremity (LE) outcomes.

|  | **LE Early Responder** | **LE Late Responder** |
| --- | --- | --- |
| n | 36 | 1 |
| **Demographics/Social** |  |  |
| Age | 55.5 [52.8, 61.3] | 70.0 [70.0, 70.0] |
| Male | 35 (97.2) | 1 (100.0) |
| Non-White Race | 6 (16.7) | 0 ( 0.0) |
| Hispanic Ethnicity | 5 (13.9) | 1 (100.0) |
| Alcohol use | 29 (80.6) | 1 (100.0) |
| Smoking |  |  |
| Former | 11 (30.6) | 1 (100.0) |
| Never or rare | 19 (52.8) | 0 ( 0.0) |
| Current | 6 (16.7) | 0 ( 0.0) |
| **Comorbidities/Medications** |  |  |
| HIV | 19 (52.8) | 0 ( 0.0) |
| VACS | 18.0 [12.0, 24.0] | 33.0 [33.0, 33.0] |
| Pre-frail | 24 (66.7) | 1 (100.0) |
| SPPB < 12 | 5 (13.9) | 0 ( 0.0) |
| Sarcopenia | 4 (11.1) | 0 ( 0.0) |
| Hypertension | 17 (47.2) | 1 (100.0) |
| Hyperlipidemia | 24 (66.7) | 1 (100.0) |
| Diabetes | 5 (13.9) | 0 ( 0.0) |
| Antihypertensives | 17 (47.2) | 1 (100.0) |
| Statins | 19 (52.8) | 1 (100.0) |
| **Randomization to HIT** | 17 (47.2) | 1 (100.0) |

**Supplemental Table S3.** Characteristics of early and late responders by upper extremity (UE) outcomes.

|  | **UE Early Responder** | **UE Late Responder** |
| --- | --- | --- |
| n | 23 | 9 |
| **Demographics/Social** |  |  |
| Age | 58.0 [53.0, 62.0] | 55.0 [53.0, 67.0] |
| Male | 22 (95.7) | 8 ( 88.9) |
| None-White Race | 6 (26.1) | 2 ( 22.2) |
| Hispanic Ethnicity | 3 (13.0) | 3 ( 33.3) |
| Alcohol use | 17 (73.9) | 9 (100.0) |
| Smoking |  |  |
| Former | 9 (39.1) | 3 ( 33.3) |
| Never or rare | 12 (52.2) | 5 ( 55.6) |
| Current | 2 ( 8.7) | 1 ( 11.1) |
| **Comorbidities/Medications** |  |  |
| HIV | 12 (52.2) | 3 ( 33.3) |
| VACS | 12.0 [12.0, 24.0] | 12.0 [12.0, 27.0] |
| Pre-frail | 15 (65.2) | 5 ( 55.6) |
| SPPB < 12 | 3 (13.0) | 2 ( 22.2) |
| Sarcopenia | 1 ( 4.3) | 0 ( 0.0) |
| Hypertension | 6 (26.1) | 6 ( 66.7) |
| Hyperlipidemia | 11 (47.8) | 5 ( 55.6) |
| Diabetes | 5 (21.7) | 0 ( 0.0) |
| Antihypertensives | 6 (26.1) | 6 ( 66.7) |
| Statins | 8 (34.8) | 4 ( 44.4) |
| **Randomization to HIT** | 13 (56.5) | 6 ( 66.7) |

**Supplemental Table S4**. Baseline demographics by HIV status.

|  | **HIV +** | **HIV -** |
| --- | --- | --- |
| n | 27 | 29 |
| **Demographics/Social** |  |  |
| Age (mean [SD]) | 57.22 (5.87) | 60.14 (6.84) |
| Male | 25 (92.6) | 27 (93.1) |
| Non-White Race | 8 (29.6%) | 3 (10.3%) |
| Hispanic Ethnicity | 4 (14.8) | 4 (13.8) |
| Alcohol use | 20 (74.1) | 24 (82.8) |
| Smoking |  |  |
| Former | 13 (48.1%) | 9 (31.0%) |
| Never or rare | 10 (37.0) | 17 (58.6) |
| Current | 4 (14.8) | 3 (10.3) |
| **Comorbidities/Medications** |  |  |
| VACS (median [IQR]) | 24.00 [14.50, 28.00] | 12.00 [12.00, 22.00] |
| Pre-frail | 21 (77.8) | 15 (51.7) |
| SPPB < 12 | 10 (37.0) | 3 (10.3) |
| Sarcopenia | 4 (14.8) | 1 ( 3.4) |
| Hypertension | 14 (51.9) | 12 (41.4) |
| Hyperlipidemia | 14 (51.9) | 17 (58.6) |
| Diabetes | 2 ( 7.4) | 4 (13.8) |
| Antihypertensives | 14 (51.9) | 12 (41.4) |
| Statins | 13 (48.1) | 12 (41.4) |

Results presented as n(%) unless otherwise stated.

**Supplemental Table S5**. Exercise adherence and adverse events over the course of the trial.

| Outcome | Overall adherence | Adherence week 0-12 | Adherence week 0-13 |
| --- | --- | --- | --- |
| **Cardiovascular** | | | |
| Responder | 88.9 [84.0, 91.7] | 91.7 [90.3, 93.1] | 88.9 [77.8, 90.3] |
| Early Responder | 87.5 [80.3, 91.7] | 90.3 [88.2, 94.4] | 83.3 [71.5, 88.9] |
| Late Responder | 88.2 [86.1, 91.7] | 93.1 [91.0, 95.1] | 83.3 [79.2, 87.5] |
| Non-responder | 81.9 [80.6, 88.9] | 91.7 [88.9, 94.4] | 75.0 [69.4, 86.1] |
| **Lower Extremity** | | | |
| Responder | 82.6 [77.8, 89.6] | 91.7 [85.4, 94.4] | 77.8 [66.6, 88.9] |
| Early Responder | 88.9 [81.9, 91.7] | 91.7 [88.9, 94.4] | 86.1 [75.0, 88.9] |
| Late Responder | 86.1 [86.1, 86.1] | 97.2 [97.2, 97.2] | 75.0 [75.0, 75.0] |
| Non-responder | 87.5 [86.1, 91.7] | 91.7 [91.7, 94.4] | 83.3 [79.2, 90.3] |
| **Upper Extremity** | | | |
| Responder | 87.5 [82.6, 91.7] | 91.7 [88.9, 94.4] | 83.3 [76.4, 88.9] |
| Early Responder | 84.7 [79.9, 90.3] | 88.9 [88.9, 93.1] | 80.6 [69.4, 88.9] |
| Late Responder | 91.7 [87.5, 93.1] | 91.7 [91.7, 94.4] | 88.9 [83.3, 94.4] |
| Non-responder | 87.5 [87.5, 87.5] | 91.7 [91.7, 91.7] | 83.3 [83.3, 83.3] |
| **Lean Mass** | | | |
| Responder | 87.5 [81.6, 91.7] | 91.7 [88.9, 94.4] | 83.3 [74.3, 88.9] |
| Non-responder | 89.6 [86.8, 91.7] | 91.7 [88.9, 94.4] | 88.9 [79.9, 91.7] |
| Negative responder | 84.7 [75.0, 88.9] | 91.7 [88.9, 93.1] | 77.8 [59.7, 86.1] |

Adherence is presented as median [IQR].

**Supplemental Table S6.** Baseline inflammatory markers and change in IL-10 by responder group and outcome.

| Outcome | Baseline hs-CRP (mg/L) | Baseline IL-6 (pg/mL) | Baseline IL-10 (pg/mL) | Change in IL-10 (pg/mL)  (Baseline-Week 24) |
| --- | --- | --- | --- | --- |
| **Cardiovascular** | | | | |
| Responder | 2.03 [0.92, 4.29] | 2.10 [1.44, 2.69] | 4.43 [2.88, 5.86] | 43.77 [-11.69, 97.92] |
| Early Responder | 1.04 [0.59, 2.22] | 1.79 [1.31, 2.31] | 4.87 [3.74, 9.09] | 25.73 [-44.68, 130.67] |
| Late Responder | 1.56 [0.97, 1.88] | 1.77 [1.53, 2.34] | 2.98 [2.66, 4.20] | 214.68 [20.33, 231.64] |
| Non-responder | 1.68 [1.16, 3.76] | 2.13 [1.88, 2.57] | 6.15 [4.24, 7.51] | 27.47 [13.38, 141.54] |
| **Lower Extremity** | | | | |
| Responder | 1.87 [0.90, 3.17] | 2.29 [1.36, 4.15] | 4.64 [3.40, 7.46] | 31.36 [-21.72, 154.38] |
| Early Responder | 1.50 [0.79, 2.61] | 1.94 [1.40, 2.34] | 4.51 [3.44, 6.11] | 42.18 [-16.07, 129.77] |
| Late Responder | 1.70 [1.70, 1.70] | 1.81 [1.81, 1.81] | 17.69 [17.69, 17.69] | -40.50 [-40.50, -40.50] |
| Non-responder | 1.07 [0.96, 1.98] | 2.10 [1.55, 2.40] | 3.43 [2.76, 4.91] | 67.53 [40.11, 197.88] |
| **Upper Extremity** | | | | |
| Responder | 1.67 [1.05, 2.38] | 2.05 [1.43, 2.53] | 4.03 [2.86, 5.16] | 43.77 [-28.72, 173.09] |
| Early Responder | 1.46 [0.81, 3.67] | 2.10 [1.62, 2.64] | 5.48 [3.84, 8.06] | 34.75 [0.64, 123.98] |
| Late Responder | 1.12 [0.81, 2.46] | 1.54 [1.31, 1.59] | 4.76 [4.09, 5.21] | 78.86 [-11.69, 128.64] |
| Non-responder | 0.34 [0.34, 0.34] | 1.78 [1.78, 1.78] | 11.43 [11.43, 11.43] | -63.14 [-63.14, -63.14] |
| **Lean Mass** | | | | |
| Responder | 1.67 [1.05, 2.80] | 2.04 [1.40, 2.61] | 4.49 [2.83, 7.14] | 50.57 [-34.22, 212.56] |
| Non-responder | 1.44 [0.76, 2.37] | 1.77 [1.30, 2.42] | 4.37 [3.15, 5.12] | 23.57 [-11.69, 116.62] |
| Negative responder | 1.06 [0.71, 3.18] | 2.00 [1.80, 3.47] | 4.54 [4.12, 9.99] | 43.78 [-35.14, 121.09] |

Results are presented as median [IQR]. Abbreviations: hsCRP, highly sensitive C-reactive protein; I-FABP, intestinal fatty acid binding protein; IL, interleukin; IQR, interquartile range; sCD14, soluble CD14; sTNFR, soluble tumor necrosis factor receptor; TNF-α, tumor necrosis factor-α.

**Supplemental Table S7**. Acute inflammatory responses at baseline by responder group.

| Outcome | IL-6 (pg/mL) | sTNFR (pg/mL) | TNF-α (pg/mL) |
| --- | --- | --- | --- |
| **Cardiovascular** | | | |
| Responder | 345.00 [243.15, 472.12] | 113400.00 [79582.50, 130492.50] | 102.75 [75.30, 118.35] |
| Early Responder | 281.40 [225.60, 460.99] | 108165.00 [90165.00, 138525.00] | 91.95 [71.10, 127.43] |
| Late Responder | 318.00 [218.36, 378.64] | 99915.00 [75750.00, 118897.50] | 76.35 [47.92, 100.28] |
| Non-responder | 436.95 [304.95, 632.10] | 113220.00 [97125.00, 118905.00] | 123.00 [64.65, 142.65] |
| **Lower Extremity** | | | |
| Responder | 355.12 [241.09, 567.75] | 102465.00 [81408.75, 114386.25] | 77.55 [61.84, 127.91] |
| Early Responder | 322.05 [263.89, 463.35] | 113310.00 [82987.50, 131463.75] | 102.75 [77.18, 129.23] |
| Late Responder | 245.40 [245.40, 245.40] | 153225.00 [153225.00, 153225.00] | 157.50 [157.50, 157.50] |
| Non-responder | 289.50 [229.87, 399.90] | 114360.00 [96367.50, 117615.00] | 74.55 [60.60, 88.20] |
| **Upper Extremity** | | | |
| Responder | 336.90 [262.35, 577.58] | 114330.00 [93052.50, 131475.00] | 101.93 [74.33, 124.24] |
| Early Responder | 307.20 [262.58, 455.48] | 107805.00 [80002.50, 119317.50] | 89.10 [66.60, 122.55] |
| Late Responder | 236.85 [169.05, 345.00] | 107265.00 [81345.00, 120240.00] | 96.60 [69.00, 116.25] |
| Non-responder | 514.65 [514.65, 514.65] | 244020.00 [244020.00, 244020.00] | 96.60 [69.00, 116.25] |
| **Lean Mass** | | | |
| Responder | 285.00 [225.60, 358.35] | 107535.00 [81412.50, 119021.25] | 77.70 [62.10, 120.38] |
| Non-responder | 322.80 [254.36, 503.03] | 118185.00 [111731.25, 147390.00] | 102.08 [82.76, 116.92] |
| Negative responder | 514.65 [376.72, 579.90] | 96705.00 [84435.00, 122197.50] | 123.00 [87.08, 144.68] |

Results are presented as the median area under the curve [IQR]. Abbreviations: hsCRP, highly sensitive C-reactive protein; I-FABP, intestinal fatty acid binding protein; IL, interleukin; IQR, interquartile range; sCD14, soluble CD14; sTNFR, soluble tumor necrosis factor receptor; TNF-α, tumor necrosis factor-α.


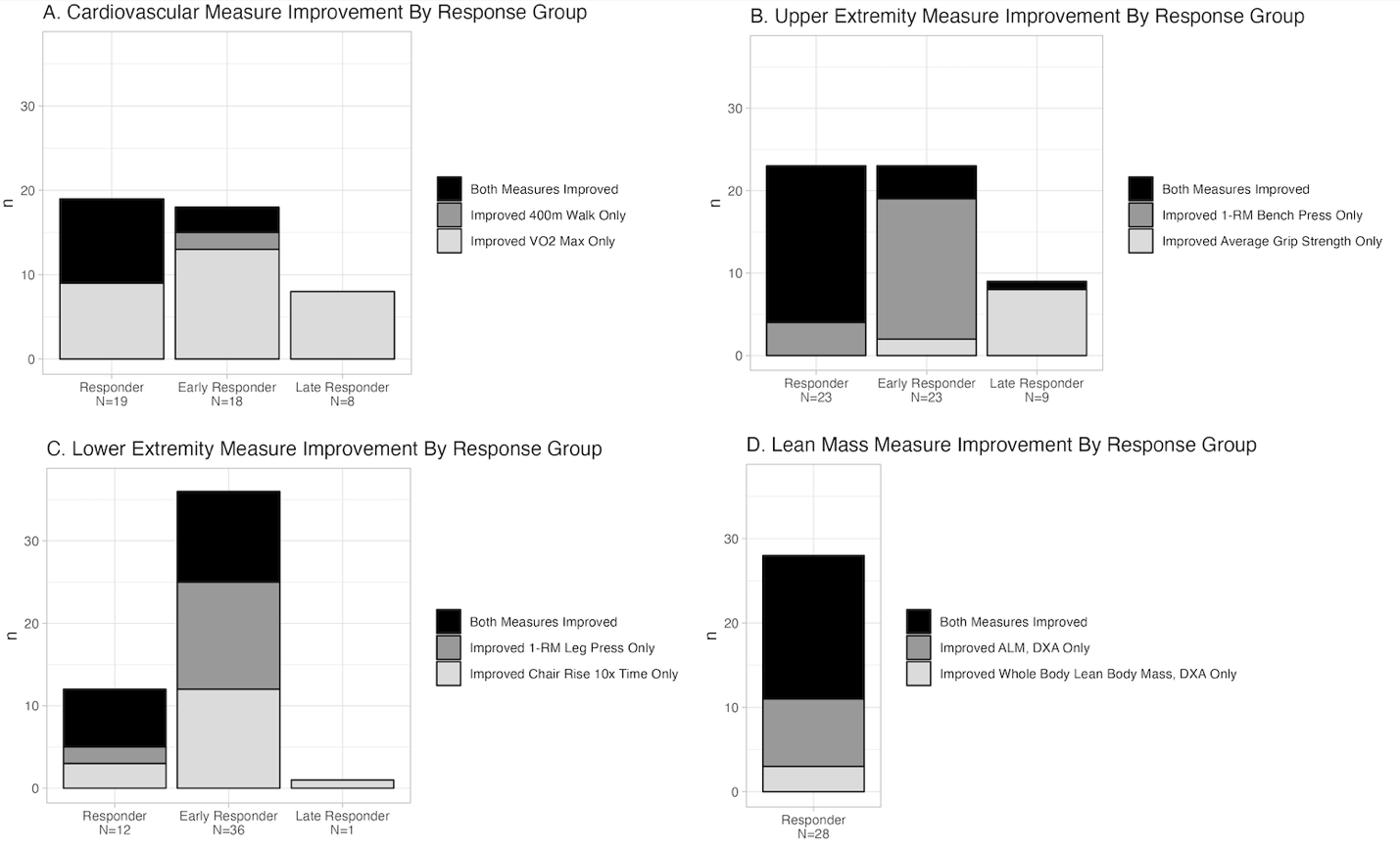


**Supplemental Fig. S1** Breakdown of participants who improved in only one outcome (n). A = cardiovascular, B = upper extremity, C = lower extremity, D = lean mass.
